# Targeting Glycolytic Plasticity to Overcome Therapy Resistance in Cancer Stem Cells: Mechanisms and Clinical Perspectives

**DOI:** 10.3390/cells15121107

**Published:** 2026-06-18

**Authors:** Jiaxin Huang, Xinyu Yang, Feiyu Li, Xinyu Li, Hao Wei, Muyao Li

**Affiliations:** 1 School of Medical and Life Sciences, Chengdu University of Traditional Chinese Medicine, Chengdu 611137, China; 19181957709@163.com (J.H.); 18728489852@163.com (F.L.); 18050394729@163.com (X.L.); 15378420085@163.com (H.W.); 2 School of Pharmacy, Chengdu University of Traditional Chinese Medicine, Chengdu 611137, China; 13678172806@163.com

**Keywords:** cancer stem cells, metabolic plasticity, glycolysis, epigenetic regulation, therapeutic resistance, tumor microenvironment

## Abstract

Cancer stem cells (CSCs) constitute a resilient tumor subpopulation responsible for multidrug resistance, metastasis, and clinical relapse. A cardinal hallmark of these cells is profound metabolic plasticity. This dynamic defense mechanism facilitates rapid shifts between glycolysis, oxidative phosphorylation (OXPHOS), and alternative nutrient catabolism, enabling CSCs to bypass microenvironmental constraints. This review delineates how glycolytic adaptation functions as a primary driver of therapy resistance within the CSC niche. We dissect the regulatory triad controlling these metabolic shifts, which includes rate-limiting enzymes, epigenetic and epitranscriptomic remodeling, and master transcription factors. Glycolytic reprogramming transcends bioenergetics by acting as a metabolic signaling node. It integrates with the epithelial–mesenchymal transition (EMT) program, autophagic pathways, and the immunosuppressive tumor microenvironment (TME) to fortify CSC survival. We appraise emerging therapeutic interventions targeting these metabolic vulnerabilities. Strategies focus on optimizing small-molecule inhibitors, nanotechnology-enabled delivery systems, and immunometabolic combination regimens. This review establishes a conceptual framework for precision interventions aimed at disrupting CSC plasticity, overcoming therapeutic resistance, and preventing tumor recurrence.

## 1. Introduction

**Figure 1:**
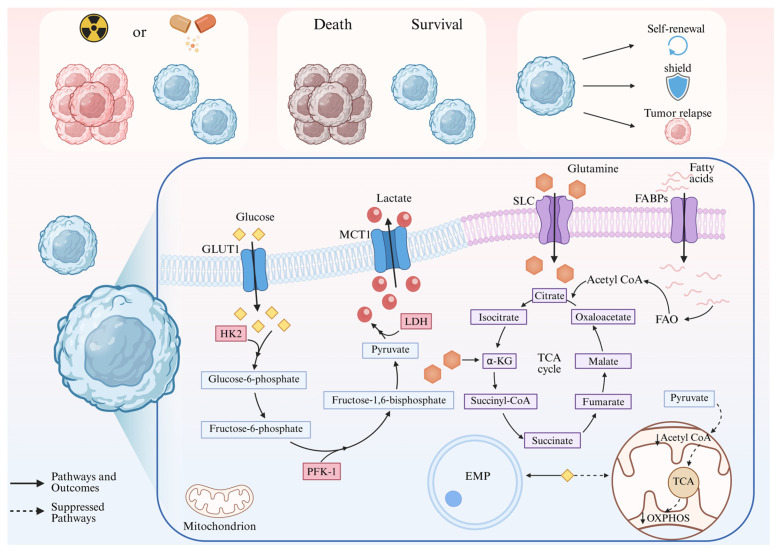
Glycolytic reprogramming in CSCs. CSCs exhibit heightened glycolytic activity compared to differentiated tumor cells, even under normoxic conditions, a phenomenon known as the Warburg effect. This schematic illustrates the metabolic flux of glucose, highlighting the commitment step catalyzed by HK2 and the conversion of pyruvate to lactate. This metabolic reprogramming robustly supports CSC self-renewal and therapeutic resistance. (Abbreviations: GLUT1, glucose transporter 1; HK2, hexokinase 2; PFK-1, phosphofructokinase-1; LDH, lactate dehydrogenase; MCT1, monocarboxylate transporter 1; EMP, Embden–Meyerhof–Parnas pathway; TCA cycle, tricarboxylic acid cycle; OXPHOS, oxidative phosphorylation; FAO, fatty acid oxidation; a-KG, α-Ketoglutarate; FABPs, fatty acid-binding proteins; SLC, solute carrier). Created in BioRender. Yang, X. (2026) https://BioRender.com/l9i0qfa (accessed on 16 May 2026).

CSCs represent a discrete tumor subpopulation defined by self-renewal capacity and the ability to generate heterogeneous progeny [1], driving tumor progression, relapse, and metastasis [2]. Distinct from bulk tumor cells, CSCs exhibit profound phenotypic plasticity and inherent drug resistance [3]. Therapy resistance remains a formidable challenge in clinical oncology [4], exacerbated by the dynamic plasticity of the TME during anti-neoplastic interventions [5,6]. The TME profoundly shapes the metabolic profile of CSCs [7], instigating a phenomenon termed metabolic plasticity, which operates as a critical defense mechanism against environmental and therapeutic stress. CSCs secure this survival advantage by dynamically calibrating their bioenergetic networks, shifting between glycolysis, mitochondrial respiration, and the catabolism of alternative substrates, including glutamine and lipids [4]. Emerging evidence indicates that CSCs frequently display attenuated basal OXPHOS activity and rely predominantly on glycolytic metabolism (Figure 1) [8]. This metabolic reprogramming sustains proliferation within hypoxic niches and confers the profound flexibility required to evade conventional chemoradiotherapy, which primarily targets rapidly dividing bulk populations [4,9].

Recognizing this metabolic vulnerability, research has focused on developing strategies to disrupt CSCs’ glycolytic addiction [10,11]. By manipulating metabolic pathways, CSCs can meet their specific energy demands and maintain their stem cell state, driving tumor progression and treatment evasion [12]. Thus, elucidating the regulatory mechanisms of glycolysis in CSCs is vital for devising new therapeutic approaches. This review offers a detailed summary of the regulatory mechanisms of glycolysis in CSCs, focusing on how key enzymes, epigenetic modifications, and transcription factors drive multidrug resistance, and evaluating emerging therapeutic strategies to overcome this plasticity.

## 2. Metabolic Heterogeneity and Adaptability in CSCs

Rather than adhering to a monolithic energy blueprint, CSC survival relies on a dual strategy of profound metabolic heterogeneity and dynamic plasticity [13]. Across distinct malignancies, CSCs exhibit remarkable inter- and intra-tumoral variance, a baseline divergence that renders broad-spectrum inhibitors largely ineffective [14]. While mapping these cancer-specific vulnerabilities offers a critical rationale for tailored therapies, CSCs further complicate treatment by actively shifting between glycolytic and oxidative states. It is this temporal adaptability to microenvironmental fluctuations and therapeutic stress that ultimately drives acquired resistance and disease progression [15].

### 2.1. Cancer-Specific Glycolytic Profiles

To establish a structured clinical baseline, this underlying metabolic variance is analyzed across two complementary dimensions.

#### 2.1.1. Inter-Tumoral Heterogeneity

The primary tissue origin and anatomical system profoundly dictate the baseline glycolytic commitments of CSCs, as exemplified by malignancies of the neurological and respiratory systems. In glioblastoma (GBM), CSCs display a pronounced glycolytic phenotype characterized by the overexpression of glucose transporters and glycolytic enzymes. This neurological malignancy showcases a state of absolute glycolytic addiction, where hyperactive glucose consumption serves as a primary bioenergetic engine to sustain cell-intrinsic survival while concurrently creating a distinct, organ-specific metabolic dependency [16]. Non-small-cell lung cancer (NSCLC) demonstrates a distinct metabolic architectures. Spatial transcriptomics reveals that ALDH+ tumor stem cell subpopulations possess unique epigenetic regulatory networks [17]. Rather than utilizing glucose in isolation, the glycolytic behavior of NSCLC CSCs is highly collaborative and extrinsic, forcefully shaping the surrounding stromal microenvironment and inducing broad metabolic reprogramming in adjacent non-tumor cell lineages to reinforce the tumor’s immune evasion network [18]. Characterizing these two distinct modalities illustrates that different organ systems force CSCs to adopt completely divergent glycolytic survival strategies.

#### 2.1.2. Intra-Tumoral Heterogeneity

Striking metabolic stratification and temporal plasticity exist within the same malignancy, particularly in reproductive and endocrine-related cancers. Breast cancer exhibits subtype-specific metabolic dependencies; basal-like subtypes rely predominantly on glycolysis, whereas luminal subtypes engage OXPHOS more extensively [19]. This metabolic divergence mirrors distinct cellular origins and molecular ontogenies. This pattern of metabolic stratification extends to ovarian cancer, which harbors heterogeneous metabolic subpopulations: specific CSC pools rely on hardwired glycolytic pathways for rapid expansion, whereas others flexibly execute oxidative pathways to preserve cellular redox homeostasis under nutrient deprivation [20]. Further exemplifying this intra-tumoral diversity, prostate cancer presents striking metabolic shifts during disease progression; castration-resistant prostate cancer (CRPC) subpopulations frequently rely heavily on enhanced glycolysis and lipid synthesis, metabolically diverging from androgen-dependent lineages that maintain distinct bioenergetic profiles [21].

This underlying complexity underscores the necessity for precision therapeutic modalities tailored to cancer-type-specific metabolic vulnerabilities, rendering broad-spectrum strategies obsolete. Mapping this baseline metabolic heterogeneity represents a prerequisite for deciphering the dynamic adaptations of CSCs under therapeutic stress.

### 2.2. Glycolysis–OXPHOS Dynamic Switch

The widespread reliance on aerobic glycolysis observed across baseline tumor profiles does not entirely dictate CSC survival strategies. Their evolutionary success is fundamentally anchored in temporal adaptability. Through profound metabolic plasticity, CSCs dynamically switch between glycolysis and OXPHOS to survive microenvironmental fluctuations, ultimately driving spatiotemporal intra-tumoral heterogeneity during disease progression [3,4]. Beyond satisfying bioenergetic and biosynthetic requirements, this metabolic agility actively orchestrates lineage commitment via metabolic signaling and epigenetic remodeling [12]. Indeed, these dual metabolic states confer distinct survival advantages, enabling CSCs to navigate spatial and temporal shifts within the tumor niche. Glycolytic dominance facilitates survival within hypoxic niches and supplies critical biosynthetic precursors to sustain rapid proliferation. Glycolysis-derived lactate acidifies the microenvironment, suppressing antitumor immunity and modulating gene expression through epigenetic mechanisms, notably histone lactylation [22]. In contrast, the OXPHOS-dependent phenotype maximizes the efficiency of nutrient-derived energy production, a state frequently triggered by glucose deprivation or therapeutic stress [23]. For example, CD44+CD117+ ovarian CSCs activate fatty acid β-oxidation and the pentose phosphate pathway to preserve redox homeostasis during glucose starvation [24]. This bidirectional plasticity underpins acquired therapy resistance. Conventional interventions frequently provoke compensatory metabolic switching, culminating in treatment failure [15]. Radiotherapy, by suppressing glycolysis via mTOR-HK2 complex formation, actively drives a compensatory shift toward mitochondrial respiration in surviving CSC populations [25]. Platinum-based chemotherapeutics exert a parallel selective pressure, enriching OXPHOS-dependent CSCs and elevating the risk of disease recurrence [26].

## 3. Molecular Drivers of Glycolytic Plasticity in Drug Resistance

**Figure 2:**
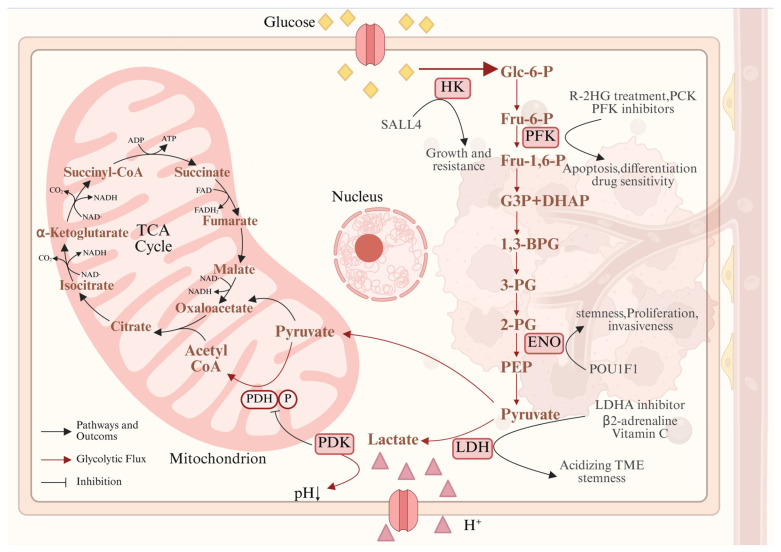
Pharmacological regulatory network of glycolysis in CSCs. This schematic illustrates the pivotal role of the glycolytic cascade in CSCs and the targeted regulation of key metabolic enzymes by diverse pharmacological compounds. The modulation of critical enzymatic hubs profoundly dictates CSC biology. Transcriptionally, SALL4 upregulates HK expression to enhance CSC therapeutic resistance, whereas specific inhibitors like R-2HG target PFK to re-sensitize CSCs to chemotherapy. Additionally, POU1F1 transcriptionally regulates ENO1 to govern glycolytic flux. Metabolically, Vitamin C modulates LDH activity, altering lactate production and remodeling the tumor microenvironment, which subsequently impairs CSC survival, migration, and invasive capacities. Collectively, these findings underscore the indispensability of glycolysis in CSC maintenance and highlight novel therapeutic vulnerabilities. (Abbreviations: SALL4, spalt-like transcription factor 4; R-2HG, R-2-hydroxyglutarate; PCK, phosphoenolpyruvate carboxykinase; TME, tumor microenvironment.) Created in BioRender. Yang, X. (2026) https://BioRender.com/l9i0qfa (accessed on 16 May 2026).

While CSCs exhibit transient metabolic plasticity, such as shifting toward OXPHOS under therapeutic stress (as discussed in Section 2.2), aerobic glycolysis remains their fundamental baseline for sustained self-renewal and rapid biosynthesis [27]. To re-establish and maintain this glycolytic dominance in hostile microenvironments, CSCs rely on a highly coordinated molecular network [28]. Key metabolic enzymes function as the core enzymatic engines and primary pharmacological targets within this network (Figure 2). Operating alongside these hubs, complex epigenetic modifiers and master transcription factors orchestrate dynamic metabolic switching to drive therapeutic resistance fundamentally.

### 3.1. Key Metabolic Enzymes as Gatekeepers of Resistance

#### 3.1.1. Hexokinase (HK)

HK2, which catalyzes glucose phosphorylation, is frequently overexpressed in cancer as a downstream consequence of phosphoinositide 3-kinase/protein kinase B (PI3K/AKT) pathway activation. This upregulation drives glycolytic flux and maintains CSC stemness [29]; clinically, HK2 is highly expressed in multiple malignancies and strongly correlates with tumor progression and poor prognosis [30]. Beyond its canonical metabolic role, HK2 acts as a pivotal regulator by facilitating acetyl-CoA accumulation, which drives the transcription of Acyl-CoA synthetase long-chain family member 4 (ACSL4) through mediating histone H3K27 acetylation via the EP300/NCOA3/SP1 complex. Subsequently, ACSL4 promotes fatty acid β-oxidation (FAO), supplying the bioenergetic and metabolic requirements essential for maintaining CSC self-renewal capacity [31]. Independent of this indirect cascade, the extramitochondrial fraction of HK2 exercises direct, non-metabolic regulatory control by physically binding the canonical CSC marker CD133 and recruiting the deubiquitinase ubiquitin-specific protease 11 (USP11) to abrogate its proteasomal degradation [32], further cementing its position in CSC plasticity and survival. Although the roles of HK2 in CSC biology have been extensively investigated, the specific functions and mechanistic contributions of other HK isoforms in CSCs remain poorly characterized and warrant further investigation [33].

#### 3.1.2. Phosphofructokinase (PFK)

PFK-1 catalyzes the conversion of fructose-6-phosphate to fructose-1,6-bisphosphate, a process dynamically regulated by the ATP/AMP ratio that represents the committed, rate-limiting step of glycolysis. In cancer cells, oncogenic signaling drives the overexpression of 6-phosphofructo-2-kinase/fructose-2,6-bisphosphatase 3 (PFKFB3), a critical kinase that synthesizes the potent allosteric activator fructose-2,6-bisphosphate (F-2,6-BP) [34]. This upregulation alleviates metabolic inhibition and markedly enhances glycolytic flux [35]. Elevated PFK enzymatic activity significantly correlates with chemotherapy resistance and enhanced tumorsphere-forming capacity in malignant pleural mesothelioma [36]. Targeted suppression of PFK activity, particularly through the inhibition of critical upstream activators like PFKFB3, has been shown to abrogate the proliferation and stemness of CSCs, as prominently evidenced in colorectal models [37].

Recent evidence has further elucidated an atypical regulatory mechanism involving protein–protein interactions: the mitochondrial membrane protein voltage-dependent anion channel 2 (VDAC2) physically associates with the platelet-type phosphofructokinase (PFKP) isoform, anchoring it to the mitochondrial membrane in glioma cells. This spatial sequestration prevents PFKP release into the cytoplasm, thereby restraining glycolytic activity. Upon VDAC2 downregulation or ablation, PFKP dissociates from the mitochondria and drives cytoplasmic glycolysis. This metabolic shift induces non-stem tumor cells (NSTCs) to acquire glioma stem cell (GSC) characteristics via augmented glucose uptake and lactate production. Reciprocally, overexpression of VDAC2 attenuates GSC glycolysis and compromises stemness [38]. The sustained hyperactivation of aerobic glycolysis, driven by these critical enzymatic hubs, is intrinsically linked to tumor plasticity, particularly EMT, fueling the metastatic potential and multidrug resistance of CSCs [39].

#### 3.1.3. Pyruvate Dehydrogenase Kinase (PDK)

PDK inhibits mitochondrial OXPHOS by phosphorylating and inactivating pyruvate dehydrogenase (PDH), thereby diverting pyruvate toward glycolysis or anabolism [40]. In cancer, oncogenic signals upregulate PDK2/3 expression. This upregulation enhances PDH phosphorylation and inhibits mitochondrial function, driving glucose flux preferentially toward lactate production via aerobic glycolysis (the Warburg effect) [41]. This metabolic reprogramming is instrumental in maintaining CSC stemness, as evidenced by the concurrent upregulation of CSC markers and multidrug resistance genes. Following this metabolic shift, PDK1/2-driven metabolic plasticity markedly exacerbates tumor aggressiveness, encompassing phenotypes ranging from tumorsphere formation, migration, and invasion to enhanced resistance to chemotherapeutic agents like cisplatin and gemcitabine. As a critical metabolic hub, PDK1/2 integrates various upstream regulatory inputs, including transforming growth factor β1 (TGFβ1) signaling and p53 mutational status, to orchestrate the metabolic shift required for CSC maintenance and head and neck cancer (HNC) progression [42]. Targeting PDK1/2 represents a promising strategy for reversing the metabolic phenotype and chemoresistance of CSCs.

#### 3.1.4. Enolase (ENO)

In cancers including head and neck squamous cell carcinoma (HNSCC), the ENO2 isoform is abnormally overexpressed. Beyond its classical metabolic function, ENO2 drives the malignant phenotype by regulating Pyruvate Kinase isozyme M2 (PKM2) protein stability and nuclear translocation. ENO2 depletion not only promotes PKM2 degradation via the ubiquitin–proteasome pathway but also inhibits its AKT-mediated nuclear translocation, thereby blocking glycolytic flux and cyclin D1 (CCND1)-driven cell cycle progression [43].

While ENO2 drives these non-canonical signaling events, its isoform ENO1 remains a central executioner of canonical glycolytic metabolism. Upregulated ENO1 expression enhances glycolytic metabolism, supplying the bioenergetic and biosynthetic requirements of CSCs [44], in turn bolstering their proliferation and invasive capabilities [45]. ENO1 overexpression promotes gastric CSC stemness through a dual molecular mechanism: metabolically, it directly enhances glucose consumption, lactate production, and extracellular acidification, providing the energetic and metabolic substrates essential for stem-like cells through aerobic glycolysis. Concurrently, ENO1 induces the upregulation of stem cell markers (CD44, OCT4, and SOX2), enhancing self-renewal, invasion, and chemoresistance [46]. Mechanistically, acetylation of ENO1 at K89/K92/K105 enhances its RNA-binding capacity via conformational changes, a modification negatively regulated by sirtuin 2 (SIRT2). Upon RNA binding, the glycolytic function of ENO1 is inhibited, and 3-phosphoglycerate is redirected toward serine synthesis. In CSCs, abnormally high SIRT2 activity deacetylates ENO1, sustaining glycolytic flux to support stemness. Targeting this regulatory axis via pharmacological SIRT2 inhibition or by promoting ENO1-RNA binding can disrupt CSC anabolism and block glycolysis, effectively dismantling their proliferative capacity and stemness [47]. Notably, the expression of ENO1 is dynamically orchestrated by the transcription factor POU class 1 homeobox 1 (POU1F1), which acts as a key upstream driver of ENO1-mediated glycolytic reprogramming and stemness maintenance [48]. Targeting this ENO-driven metabolic hub, either by modulating POU1F1-mediated transcriptional output or by disrupting the SIRT2/ENO1 regulatory axis, represents a potent therapeutic strategy to overcome CSC chemoresistance and self-renewal capacity.

#### 3.1.5. Lactate Dehydrogenase (LDH)

CSCs rely on anaerobic glycolysis for energy supply in the hypoxic microenvironment. LDH, particularly the LDHA isoform, catalyzes the conversion of pyruvate to lactate, maintaining the glycolysis pathway and generating lactate, a key metabolic byproduct [49,50]. Lactate can enhance the proliferative capacity, anti-apoptotic potential, and drug resistance of CSCs. The oncogenic functions of LDHA include acidifying the tumor microenvironment through excessive lactate production, promoting tumor invasion, and inhibiting the anti-cancer immune response [51,52]. Chronic stress-induced β2-adrenergic signaling can upregulate the expression of LDHA, maintaining the stemness of CSCs by promoting lactate production [53]. Therapeutic targeting of LDHA activity effectively dismantles these malignant phenotypes. Intriguingly, LDHA expression is downregulated in isocitrate dehydrogenase (IDH)-mutant gliomas, which may contribute to the slower disease progression and improved prognosis observed in IDH-mutant gliomas [54].

Fundamentally, the significance of these rate-limiting enzymes extends far beyond maintaining classical bioenergetic homeostasis. The metabolic fluxes they drive, including the aberrant accumulation of lactate, directly supply the essential chemical substrates required for downstream chromatin remodeling [55]. This bidirectional metabolic–epigenetic crosstalk forms a robust feedback network that locks CSCs into a state of stemness and therapy resistance [56], naturally directing our focus toward the overarching epigenetic regulatory networks.

### 3.2. Epigenetic Control of Glycolytic Adaptation

**Figure 3:**
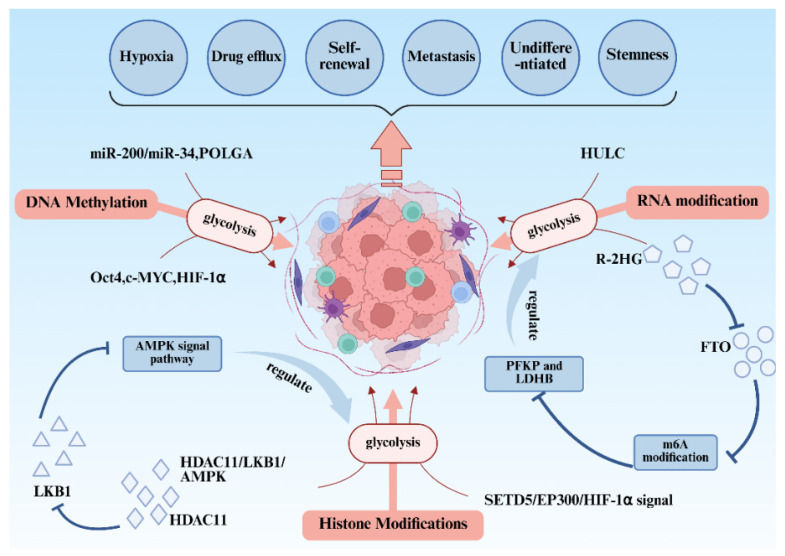
Epigenetic and epitranscriptomic regulation sustaining CSC metabolism. This diagram delineates the intricate interplay between epigenetic modifications and the glycolytic pathway in CSCs. These epigenetic layers enable CSCs to adapt to therapeutic stress, preserve stemness features, and maintain metabolic homeostasis. Specifically, DNA methylation governs the expression of critical miRNAs and transcription factors essential for CSC identity and glycolytic output. Concurrently, histone acetylation and the targeted inhibition of HDAC11 fine-tune the chromatin accessibility of metabolic genes. Epitranscriptomic marks, notably m6A modification and the FTO demethylase, dictate the stability and translation of glycolytic enzyme transcripts. The figure also highlights the therapeutic potential of targeting these epigenetic–metabolic axes using agents such as R-2HG. (Abbreviations: miR, microRNA; OCT4, octamer-binding transcription factor 4; HULC, highly up-regulated in liver cancer; FTO, fat mass and obesity-associated protein.) Created in BioRender. Yang, X. (2026) https://BioRender.com/l9i0qfa (accessed on 5 June 2026).

Epigenetic modifications represent heritable chemical alterations to DNA and chromatin that leave the underlying nucleotide sequence intact, encompassing DNA methylation, histone modifications, and epitranscriptomics [57]. These epigenetic programs constitute the architectural foundation of CSC metabolic reprogramming (Figure 3). By remodeling chromatin accessibility, they dictate the transcriptional potential required for cancer stem cells, notably breast cancer stem cell (BCSC) populations, to maintain their stemness and adaptive plasticity [58]. Growing evidence indicates that epigenetic remodeling extends beyond the tumor cell compartment to shape the TME [59], particularly through the modulation of interactions with immune cells. Therefore, exploiting epigenetic vulnerabilities in the context of tumor immunotherapy holds significant promise for the development of innovative combinatorial therapeutic strategies.

#### 3.2.1. DNA Methylation

DNA methylation acts as a fundamental epigenetic constraint that shapes the transcriptional landscape, thereby defining the metabolic plasticity of CSCs [60]. By modulating chromatin accessibility, DNA methylation programs dictate the potential for metabolic rewiring required to sustain CSC stemness [61]. In a highly synergistic manner, metabolic fluxes like serine metabolism-driven production of the universal methyl donor S-adenosylmethionine (SAM), directly couples bioenergetic states to global DNA methylation patterns, reinforcing the tumorigenic potential of CSCs [62]. At the regulatory level, high expression of DNA methyltransferases (DNMTs), including DNMT1, stabilizes the undifferentiated state by silencing specific metabolic and stemness-associated genes [63]. Pharmacological inhibition of DNMTs triggers the reactivation of these silenced genes, effectively impairing the oncogenic capacity of gastric and colorectal CSCs [64,65]. Future research remains essential to delineate the precise hierarchy through which DNA methylation orchestrates the metabolic flexibility necessary for CSC adaptation.

#### 3.2.2. Histone Modifications

Diverse histone modifications serve as pivotal epigenetic regulators in CSCs, governing self-renewal, pluripotency, proliferation, and therapeutic resistance by remodeling chromatin accessibility [66]. These epigenetic marks also target key enzymes involved in glycolysis. On the transcriptional activation front, the histone methyltransferase (HMT) SETD5 complexes with EP300 and hypoxia-inducible factor-1α (HIF-1α) in BCSCs. Specifically, SETD5 synergizes with EP300 to deposit activating histone methylation and acetylation marks across target promoters. This concerted epigenetic remodeling establishes a highly permissive chromatin conformation that robustly drives glycolytic gene transcription, ultimately sustaining BCSC stemness and metabolic activity [67]. On the transcriptional repression front, the histone deacetylase HDAC11 directly represses the expression of the tumor suppressor liver kinase B1 (LKB1). This epigenetic silencing modulates glycolysis and maintains the stemness of hepatocellular carcinoma (HCC) stem cells, neutralizing the intrinsically anti-proliferative effects of activated LKB1 [68]. Glycolytic byproducts themselves, such as lactate, can act as epigenetic modulators. Lactate can inhibit histone deacetylase activity, thereby enhancing the stem cell-like properties of CD8+ T cells and bolstering anti-tumor immunity [69]. Capitalizing on this intricately woven metabolic–epigenetic network, targeting histone modifiers emerges as a highly promising therapeutic strategy for disrupting CSC glycolytic plasticity and overcoming tumor recurrence. For instance, natural compounds have demonstrated substantial anticancer potential by modulating miRNA expression and Sirtuin activity, specifically undermining CSC maintenance and differentiation. By interfering with multiple pathways, including cell cycle regulation, DNA repair, and oxidative stress, these compounds effectively impede tumor progression [70].

#### 3.2.3. N6-Methyladenosine (m6A) RNA Modification

M6A is the most prevalent internal RNA modification in eukaryotes, abundantly decorating both mRNAs and ncRNAs. This dynamic epigenetic mark plays a crucial role in the metabolic reprogramming of CSCs by dictating RNA stability, translation efficiency, and subcellular localization. In leukemia stem cells, metabolic inhibition of the m6A demethylase fat mass and obesity-associated protein (FTO) disrupts m6A homeostasis. The abrogation of FTO activity decreases the mRNA stability of key glycolytic transcripts (PFKP and LDHB), thereby suppressing glycolytic flux and compromising CSC bioenergetics [71]. The metabolic impact of m6A remains highly context-dependent. It conversely drive tumor cell proliferation and glycolytic gene transcription by enhancing the mRNA stability of critical upstream regulators, establishing a coordinated network between metabolism and cellular expansion [72]. In NSCLC, methyltransferase-like 3 (METTL3)-mediated m6A installation enhances the stability of specific non-coding RNAs, indirectly remodel the bioenergetic landscape. Concurrently, m6A modifications directly dictate the translation efficiency or degradation rates of glycolysis-related mRNAs via YTH N6-methyladenosine RNA-binding protein (YTHDF) family reader proteins. These multi-layered mechanisms sustain CSC self-renewal and reshape their metabolic dependencies, enabling persistent proliferation within hypoxic niches while conferring therapeutic resistance. From a translational perspective, therapeutically targeting m6A machinery represents a highly promising strategy for dismantling CSC metabolic plasticity [73].

In summary, dynamic epigenetic remodeling serves as a fundamental driver of CSC biology. DNA methylation dictates global transcriptional landscapes to sustain undifferentiated states, while histone modifications alter chromatin accessibility to coordinate self-renewal, pluripotency, and drug resistance programs. Epitranscriptomic marks, predominantly m6A, profoundly govern CSC function by fine-tuning RNA stability, splicing, and translational efficiency, thereby driving tumor initiation and progression. These findings underscore the indispensability of epigenetic and epitranscriptomic networks in CSC metabolic reprogramming, highlighting vulnerabilities that may be exploited for next-generation precision cancer therapies.

#### 3.2.4. Epigenetic Changes Induced by Metabolic Stress

Metabolic stress or therapeutic interventions can induce profound epigenetic remodeling in CSCs, modulating glycolysis and enhancing their adaptive capacity [74]. Under metabolic stress, DNMTs hypermethylate and transcriptionally silence specific tumor suppressor loci, including the thrombospondin 1 (THBS1) promoter [75]. Tumor cells adeptly exploit this targeted epigenetic silencing to bypass growth inhibition, thereby promoting aberrant extracellular matrix remodeling and accelerating tumor progression. This vividly highlights how CSCs leverage epigenetic plasticity to flexibly switch survival pathways amidst diverse microenvironmental crises. Metabolites themselves profoundly participate in this underlying epigenetic remodeling. For instance, lactate acts directly as an epigenetic donor, adding lactyl groups to histone lysine residues, generating activating marks akin to acetylation. This targeted modification selectively alters the chromatin conformation of key glycolytic genes to amplify metabolic flux. The regulatory network of lactate is multidimensional: beyond direct lactylation, it also enhances histone acetylation by expanding the intracellular Acetyl-CoA pool, thereby activating transcription of the proto-oncogene MYC in a bromodomain-containing protein 4 (BRD4)-dependent manner. This epigenetic activation ultimately reinforces the glycolytic phenotype, establishing a robust metabolic–epigenetic regulatory axis that relentlessly fuels CSC proliferation, metabolic plasticity, and stemness [76]. At the post-transcriptional level, non-coding RNAs complement this network by directly regulating metabolic transcripts, firmly locking in the glycolytic expression program to ensure sustained adaptation [77].

### 3.3. Transcription Factors Orchestrating Adaptive Glycolysis

The epigenetic modifications establish the foundational chromatin accessibility required for metabolic gene activation. Within this permissive epigenetic landscape, master transcription factors function as central signal integrators, directly translating environmental stimuli into targeted transcriptional programs [2,78]. Recent studies have increasingly highlighted the pivotal roles of transcription factors in orchestrating the metabolic reprogramming of CSCs [79]. Operating in tandem with diverse signaling cascades, these effectors collectively drive glycolytic addiction by directly upregulating critical metabolic enzymes [15]. Elucidating the intricate networks governing this metabolic plasticity is imperative for the development of next-generation precision oncology therapeutics. A comprehensive overview of the associations among these master transcription factors, glycolysis, and CSC stemness is summarized in Table 1.

#### 3.3.1. HIF

HIFs serve as paramount transcriptional regulators orchestrating cellular adaptation to low oxygen tension, directly dictating metabolic homeostasis and tumorigenesis [92].

To initiate targeted metabolic programs, diverse mechanisms converge to stabilize HIF-1α. Deubiquitinases (DUBs) directly reverse its von Hippel–Lindau (VHL)-mediated polyubiquitination. This targeted deubiquitination effectively rescues HIF-1α from destruction, thereby sustaining its metabolic transcriptional activity [93]. In parallel, the hepatocyte growth factor (HGF)/c-MET signaling cascade promotes yes-associated protein (YAP) nuclear translocation to specifically stabilize HIF-1α expression [94]. Acting upstream of these protein complexes, non-coding RNAs directly dictate HIF expression, adding crucial regulatory layers to this extensive stabilization network [95]. Stabilized HIF-1α undergoes massive nuclear accumulation to execute metabolic reprogramming by directly binding to hypoxia response elements within specific target promoters. A central downstream effector within this axis is carbonic anhydrase IX (CAIX). HIF-1α transcriptionally upregulates the CA9 gene to establish a critical rheostat for intracellular pH equilibrium. By facilitating rapid proton efflux, CAIX prevents lethal intracellular acidosis resulting from excessive glycolysis, simultaneously exacerbating an acidic extracellular niche that inherently promotes tumor invasion and stem cell characteristics [96]. Concurrently, stabilized HIF-1α directly induces core pluripotency markers, including NANOG, OCT4, and SOX2, while enhancing HK2 activity to fuel stemness and glycolysis in pancreatic CSCs [94].

Complementing canonical biochemical cascades, the tumor microenvironment imposes dynamic modulation on HIF activity. For instance, in colorectal CSCs, the 3D extracellular matrix can enhance glycolysis and reprogram HIF-1α-induced transcriptional networks by mediating the binding and release of F-actin. This structural dynamic triggers the degradation of PFK through tripartite motif-containing 11 (TRIM11), thereby amplifying CSC stemness [97].

#### 3.3.2. c-MYC

The proto-oncogene c-MYC encodes a pleiotropic transcription factor that drives cell cycle progression, thereby dictating cell growth and differentiation trajectories [98]. Within the tumor microenvironment, paracrine signaling from gastric cancer mesenchymal stem cells (GCMSCs) primarily induces the upregulation of c-MYC in gastric cancer cells. Subsequently, the accumulated c-MYC, acting as a master transcription factor, directly transactivates the downstream HK gene, thereby accelerating glycolytic flux and enhancing metastatic proliferation [86]. While GCMSCs fuel this c-MYC-driven axis, intrinsic regulators like caveolin-1 (Cav-1) actively suppress it. In BCSCs, Cav-1 facilitates VHL-mediated ubiquitination and degradation of c-MYC, stifling self-renewal capacity and aerobic glycolysis [99]. Disruption of this Cav-1 shield hyperactivates glycolysis at the expense of mitochondrial respiration, locking the into a Warburg phenotype that sustains the BCSC population.

Beyond direct transactivation of glycolytic effectors, c-MYC transcriptionally represses miR-192-5p to establish a robust positive feedback loop in HCSCs, persistently amplifying glycolytic dependence [82]. The impact of this miR-192-5p deficiency extends beyond tumor cells by facilitating critical paracrine crosstalk with the surrounding stroma. Driven by this hyperactive glycolysis, excessive lactate extruded by HCC cells activates the lactate/monocarboxylate transporter 1 (MCT1)/n-myc downstream-regulated gene 3 (NDRG3)/phosphorylated extracellular signal-regulated kinase (pERK) signaling cascade in adjacent non-tumor cells, nurturing a highly invasive and stem-like tumor microenvironment [82].

#### 3.3.3. p53

The transcription factor p53 functions as the canonical guardian of the genome, exerting profound control over cellular metabolism to restrict stemness and inhibit tumorigenesis [100]. To execute its metabolic regulatory function, p53 transcriptionally induces the tp53-induced glycolysis and apoptosis regulator (TIGAR) gene to potently suppress glycolytic flux. Dismantling this regulatory nexus through TP53 loss-of-function mutations unleashes uncontrolled glycolysis, enabling CSCs to survive under extreme hypoxic constraints [101]. Under hypoxia, HIF-1α is stabilized and acts as an upstream regulator to activate the transcription factor paired box 3 (PAX3). PAX3 specifically binds to the TP53 promoter region and downregulates p53 expression through transcriptional repression. The ensuing loss of p53 relieves the inhibition of glycolysis and forcefully shifts cell metabolism toward anaerobic glycolysis, providing energy for cancer cell proliferation and enhancing metastatic capacity [102]. Decoding this intricate HIF-1α/PAX3/p53 circuitry not only illuminates the survival strategies of hypoxic CSCs but also unveils crucial target vulnerabilities for disrupting their metabolic plasticity and overcoming adaptive resistance.

#### 3.3.4. Other Transcription Factors

The nuclear factor-kappa B (NF-κB) transcription factor family is frequently hyperactivated in CSCs, driving proliferation, metastasis, stemness, and therapeutic resistance. In ovarian cancer, pharmacological inhibition of PFKFB3 has been shown to curtail both glycolytic flux and NF-κB signaling [103]. NF-κB acts as a critical signaling hub where chronic inflammatory cues converge with metabolic reprogramming. This functional integration enables NF-κB to coordinate the bioenergetic requirements necessary to sustain the cancer stem cell niche effectively [104]. Beyond canonical pathways, ETS variant 4 (ETV4) is robustly implicated in driving malignant phenotypes. In BCSCs, ETV4 transcriptionally upregulates genes involved in glycolytic metabolism and activates the Sonic Hedgehog (SHH) signaling pathway, thereby sustaining BCSC stemness [88]. Beyond glycolytic reliance, overexpression of PGC-1α drives a metabolic shift toward mitochondrial OXPHOS in CSCs. A complex feedback loop exists between these factors: while activated HIF-1α upregulates PGC-1α expression, reciprocal activation of PGC-1α promotes HIF-1α protein degradation [90]. The precise molecular mechanisms governing this reciprocal crosstalk remain an active area of investigation.

Master transcription factors therefore emerge as indispensable linchpins that couple metabolic reprogramming with the maintenance of CSC traits. Functioning as central hubs, these key regulators integrate microenvironmental cues and intracellular signaling networks to dictate the bioenergetic landscape of CSCs. Unraveling the intricate regulatory networks governed by these transcription factors will unveil novel metabolic vulnerabilities, offering promising therapeutic strategies for eradicating CSCs.

## 4. Integrated Mechanisms of Glycolysis-Mediated Resistance

The persistence of CSCs during therapeutic intervention extends beyond cell-autonomous metabolic rewiring. Glycolysis functions as a pivotal hub, coupling bioenergetic output with broader survival networks, including remodeling of the TME and dynamic crosstalk with stress-response cascades.

### 4.1. Glycolysis and the Immunosuppressive TME

In tandem with securing cell-intrinsic survival, glycolytic reprogramming profoundly orchestrates the tumor immune microenvironment. Key glycolytic hubs confer resistance to T-cell-mediated cytotoxicity. Targeted ablation of glucose transporter 1 (GLUT1) triggers metabolic rewiring and robust reactive oxygen species (ROS) accumulation. This ROS burst enhances tumor necrosis factor-α (TNF-α)-mediated cell death by engaging the classical Fas-associated protein with death domain (FADD)/caspase-8 apoptotic axis, thereby thwarting tumor immune evasion [105]. During hyperactive aerobic glycolysis, high glucose levels promote HK2 dissociation from mitochondria and translocation to the cytoplasm, where it binds and phosphorylates inhibitor of nuclear factor kappa-B alpha (IκBα) at residue T291. This enhances the binding of IκBα to μ-calpain, leading to IκBα degradation, the release of NF-κB, and ultimately the upregulation of programmed death-ligand 1 (PD-L1) expression, facilitating robust immune escape [106].

This metabolic–immune crosstalk extends beyond cellular interactions to deeply remodel the tissue vascular niche. Vascular endothelial growth factor (VEGF) stimulation in endothelial cells accelerates glycolytic flux and lactate production, which in turn elevates global histone H3K9 lactylation (H3K9la) to drive transcription of angiogenesis-related genes. To lock in this pro-angiogenic state, glycolysis-derived lactate fuels an epigenetic positive feedback loop where H3K9la suppresses the expression of histone deacetylase 2 (HDAC2), a known lactyl-eraser [107]. This chaotic vascular network exacerbates local hypoxia and physically impedes the infiltration of cytotoxic T cells, thereby fortifying the immunosuppressive niche [108].

### 4.2. Metabolic Rewiring Fuels the EMT

Metabolic reprogramming, particularly aerobic glycolysis, is inextricably linked to the EMT program. Glycolytic hyperactivation not only supplies the bioenergetic and biosynthetic resources required for cell motility but also generates excess lactate [109]. This lactate acts as a critical signaling molecule, directly stabilizing EMT-inducing transcription factors (EMT-TFs), such as Snail, Twist, and ZEB1, via TGF-β signaling crosstalk. Beyond this canonical lactate-driven axis, alternative metabolic branches synergistically fuel EMT. For instance, in HCC, overexpression of glucose-6-phosphate dehydrogenase (G6PD), the rate-limiting enzyme of the pentose phosphate pathway, activates signal transducer and activator of transcription 3 (STAT3) signaling, thereby inducing EMT and driving cellular migration and invasion [110]. This metabolic–mesenchymal coupling is similarly conserved in pancreatic cancer, where hypoxic conditions stabilize HIF-1α to transcriptionally upregulate liver-type glycogen phosphorylase (PYGL). PYGL catalyzes the mobilization of glycogen into glucose-1-phosphate, channeling carbon flux into the glycolytic pathway to fulfill the elevated metabolic demands of tumor cells [111]. This enhanced glycolytic flux systematically activates downstream oncogenic cascades to orchestrate the EMT program.

### 4.3. Autophagy: A Double-Edged Sword in CSC Survival and Therapeutic Response

Autophagy, a highly conserved cellular degradation and recycling process, exhibits a paradoxical, context-dependent role in tumorigenesis. As a tumor suppressor, basal autophagy safeguards genomic stability and cellular health by clearing damaged organelles and toxic protein aggregates, thereby preventing the accumulation of oncogenic mutations and maintaining metabolic homeostasis. This tumor-suppressive function is particularly evident during early-stage carcinogenesis, where autophagic deficiency in premalignant cells correlates with heightened genomic instability and malignant transformation [112]. For instance, the targeted deletion of core autophagy genes (Atg5 or Atg7) in murine models promotes spontaneous tumor formation, underscoring the essential role of autophagy in restraining early tumorigenesis [113]. Conversely, in established tumors, autophagy is frequently hijacked by malignant cells to supply essential bioenergetic and biosynthetic substrates under nutrient deprivation or metabolic stress, thereby fueling their self-renewal and proliferation [114].

The dynamic interplay between glycolysis and autophagy profoundly influences CSC plasticity and therapeutic outcomes through multifaceted mechanisms. For example, the glycolytic byproduct lactate can actively modulate autophagic flux, facilitating autophagosome formation and autophagic degradation [115]. Glycolytic activity intricately modulates autophagy via master metabolic sensors, predominantly through activation of adenosine monophosphate-activated protein kinase (AMPK) (pro-autophagic) and suppression of mammalian target of rapamycin (mTOR) (anti-autophagic) signaling cascades [116]. Notably, HK2 functions as a metabolic switch: it inhibits autophagy in nutrient-rich environments, whereas pharmacological interventions that reduce HK2 expression can trigger protective autophagy in endometrial cancer cells. The impact of autophagy on CSCs is highly context-dependent; basal autophagy sustains CSC stemness via CD44 maintenance, whereas its pharmacological hyperactivation may confer cytoprotection, and conversely, its genetic ablation (ATG5 silencing) compromises CSC viability. Upstream regulators further complicate this network; for instance, in gastric cancer, the transcription factor spalt-like transcription factor 4 (SALL4) drives HK2 transcription to augment glycolysis, thereby accelerating proliferation and invasion [117]. In prostate CSCs, glutamine (Gln) deprivation triggers ATG5-mediated autophagy via the AMPK/mTORC1-ULK1 signaling axis. Glutamate derived from autophagic degradation is converted via transamination to α-ketoglutarate (α-KG). As an essential cofactor for Jumonji C-domain (JmjC) histone demethylases, α-KG maintains an open chromatin landscape to sustain the expression of core pluripotency genes (OCT4 and SOX2). Concurrently, this glutamate pool directly fuels glutathione (GSH) synthesis, scavenging reactive ROS to preserve redox homeostasis. By integrating GSH-mediated antioxidant defense and α-KG-driven epigenetic regulation, this autophagy-dependent metabolic rewiring ultimately safeguards the bioenergetic stability and stemness properties of CSCs. Genetic inhibition of ATG5 or pharmacological blockade of autophagosome maturation disrupts these pathways, precipitating a dual crisis: excessive ROS accumulation triggers oxidative damage, while α-KG depletion impairs JmjC demethylase function and represses chromatin accessibility. Together, this metabolic imbalance and epigenetic dysregulation culminate in the loss of CSC viability and heightened radiosensitization [118].

Functioning as a central metabolic hub, aerobic glycolysis orchestrates the invasive phenotype of CSCs through bioenergetic reprogramming during EMT. Extending beyond intrinsic survival, glycolytic byproducts actively sculpt an immunosuppressive microenvironment and drive VEGF-mediated tumor angiogenesis. In parallel with this extracellular remodeling, an internal crosstalk between glycolysis and autophagy endows CSCs with the metabolic flexibility and redox stability required to withstand severe nutrient deprivation and therapeutic stress. The convergence of these intrinsic and extrinsic mechanisms forges a multidimensional signaling network that robustly supports tumor progression and acquired resistance. Elucidating this comprehensive regulatory landscape therefore provides a crucial theoretical foundation for designing next-generation strategies to dismantle the glycolysis–autophagy–stemness axis.

## 5. Therapeutic Targeting of Glycolytic Plasticity

Deciphering the molecular drivers and integrated networks governing glycolysis-mediated resistance provides a mechanistic rationale for next-generation therapeutic strategies. Given the profound addiction of CSCs to metabolic reprogramming, exemplified by the dynamic crosstalk among glycolysis, OXPHOS, and autophagy, exploiting these specific vulnerabilities offers a promising avenue for precision therapeutic intervention.

### 5.1. Inhibitors, Combination Therapies, and Emerging Delivery Systems

Recent advancements have driven a significant expansion in pharmacological strategies targeting CSCs. To systematically dismantle the metabolic homeostasis that underpins CSC survival, these emerging therapeutic modalities can be broadly stratified into three distinct domains.

#### 5.1.1. Small-Molecule Inhibitors of Metabolic Hubs

Targeting rate-limiting enzymes remains a cornerstone of CSC-directed metabolic therapy. At the distal end of glycolysis, the selective LDHA inhibitors NHI-1 and NHI-2 downregulate GLUT1 and PKM2, thereby inducing cell cycle arrest and dissipating mitochondrial membrane potential in GSCs [119]. Moving upstream in the metabolic cascade, the PDK family emerges as another highly viable vulnerability. The natural PDK4 inhibitor KIS37 suppresses pancreatic and colorectal cancer progression by attenuating PI3K/AKT/mTOR signaling and downregulating CSC markers. Broadening this blockade, combining the pan-PDK inhibitor dichloroacetate (DCA) with salinomycin profoundly suppresses hepatocellular CSC proliferation and EMT through metabolic reprogramming. Corroborating these pharmacological findings, genetic ablation of PDK1 severely impairs glycolytic flux and stemness maintenance, reinforcing the PDK family as a crucial target node [120,121]. Further up the glycolytic pathway, PFK presents a critical bottleneck. PFKP expression can be selectively altered by R-2-hydroxyglutarate (R-2HG) or modulation of the m6A demethylase FTO [71]. Exploiting intrinsic metabolic feedback, supraphysiological concentrations of citrate, an endogenous allosteric inhibitor of PFK1 and PFK2, potently suppress glycolytic flux, deplete intracellular ATP, and antagonize HIF-1α and PI3K/AKT signaling, ultimately inducing apoptosis and restoring cisplatin sensitivity [122]. To circumvent compensatory resistance, multi-target natural agents and mitochondrial disruptors are increasingly being deployed. Polyphenolic compounds like quercetin and curcumin dismantle the glycolytic cascade by concurrently inhibiting HK2, GLUT1, and LDHA, effectively starving glycolysis-addicted cancer cells [123]. Striking at the alternative energy reservoir, compounds like d-TPP specifically disrupt the mitochondrial OXPHOS machinery by competitively inhibiting the pyruvate dehydrogenase complex (PDHc) and α-ketoglutarate dehydrogenase (KGDH), leading to severe ATP depletion [124]. By systematically severing these diverse energetic lifelines, such targeted interventions force CSCs into a state of lethal metabolic stress, permanently abolishing their self-renewal capacity, as summarized in Table 2 [125].

#### 5.1.2. Nanotechnology-Enabled Delivery Systems

Conventional glycolysis inhibitors display potent preclinical anti-tumor efficacy, yet their clinical translation is constrained by unfavorable pharmacokinetic profiles, such as restricted blood–brain barrier permeability and off-target toxicities [128]. To surmount these translational barriers, recent bioengineering efforts have yielded multifunctional nanocarriers, such as the GNSs-dPG-3BP/TPP/HA nanocomposite [129]. This advanced delivery platform facilitates the mitochondria-targeted release of 3BP to specifically inhibit HK2 and trigger apoptosis, elegantly integrating spatiotemporal delivery, metabolic blockade, and photothermal ablation to eradicate CSCs. Expanding on this nanomedicine approach, other studies have investigated amphiphilic block-dendritic polymer nanoparticles (NPs) for the co-delivery of the EGFR-TKI gefitinib and YAP-siRNA, maximizing therapeutic efficacy while minimizing systemic toxicity [121]. Nevertheless, these nanomedicines face persistent biocompatibility challenges. The complex physiological architecture and metabolic heterogeneity inherent in human patients often diverge significantly from those of controlled animal models, which complicates Phase II–IV trials and restricts the clinical translation of preclinical outcomes [130,131].

#### 5.1.3. Immunometabolic Combination Therapies

The intricate interplay between CSC glycolysis and the tumor immune microenvironment (TIME) provides a compelling rationale for combination therapies. Monocarboxylate transporter 4 (MCT4) is frequently overexpressed in CSCs, making it a prime therapeutic target. Selective MCT4 inhibitors block lactate efflux, precipitating intracellular lactate accumulation and the subsequent disruption of HIF-1α/PI3K/AKT/mTOR-driven glycolytic metabolism [132]. This metabolic blockade not only downregulates core stemness factors but also alleviates extracellular acidification, thereby restoring the cytotoxic recognition capabilities of CD8+ T-cells. Crucially, highly selective MCT4 inhibitors like AZD0095 spare normal cellular metabolism, highlighting the translational potential of targeting the CSC-immune metabolic axis [133]. Building upon this immune-restoring potential, combining glycolysis inhibitors with immune checkpoint blockade (ICB) exerts profound synergistic effects via multifaceted TIME remodeling. Specifically, suppression of ATP and lactate production activates the AMPK/mTOR axis to repress stemness factors, while parallel attenuation of Wnt signaling downregulates PD-L1 and immunosuppressive cytokines [18,134]. Extending this impact to the broader immune niche, metabolic blockade abrogates lactate-mediated T-cell receptor (TCR) suppression, impedes the recruitment of MDSCs and Tregs, and upregulates MHC-I to enhance antigen presentation. When paired with ICB, which directly reverses T-cell exhaustion, these combined modalities synergistically reprogram the hostile TIME into a permissive niche, driving robust effector T-cell infiltration and durable anti-tumor immunity [135]. Current immunometabolic approaches are often limited by low clinical response rates and a high incidence of immune-related adverse events (irAEs). For instance, while anti-PD1/PD-L1 therapies yield response rates around 30% in NSCLC, anti-CTLA-4 treatments are associated with irAE rates nearing 70%. These dual challenges in efficacy and safety necessitate the discovery of novel immune checkpoints to enhance therapeutic outcomes [136].

### 5.2. Metabolic Biomarkers for Predicting Recurrence

Specific glycolytic metabolites not only provide critical mechanistic insights into tumor recurrence but also serve as robust predictive biomarkers. For instance, excessive extracellular lactate drives tumor angiogenesis via the upregulation of H3K9la in endothelial cells [137]. Intracellularly, lactate induces the lactylation of specific DNA repair proteins, thereby facilitating DNA damage repair and conferring therapeutic resistance [138]. Additionally, the oligomeric dynamics of metabolic enzymes drive glycolytic reprogramming in GSCs, exacerbating radio- and chemoresistance while serving as prognostic indicators of treatment evasion [139]. Thus, therapeutically targeting these epigenetic–metabolic axes, exemplified by targeted inhibition of PFKFB3 to abrogate angiogenesis [140,141], or blockade of CPT1A-mediated MFF succinylation to attenuate ovarian CSC stemness [142], represents the next frontier in precision oncology.

## 6. Conclusions and Future Perspectives

The metabolic adaptability of CSCs represents a highly orchestrated survival strategy rather than a passive biochemical response. Through the synergistic integration of rate-limiting enzymatic hyperactivation, precise epigenetic remodeling, and systemic transcriptional control by master regulators like HIF-1α and c-MYC, CSCs construct a robust metabolic fortress. This profound coupling of glycolytic dominance with phenotypic plasticity, particularly through the EMT program and immune-suppressive signaling, constitutes the fundamental engine of acquired therapeutic resistance and tumor relapse. Despite the therapeutic promise demonstrated in preclinical models, a substantial chasm remains in clinical translation, primarily driven by high metabolic redundancy and compensatory escape mechanisms, where CSCs rapidly shift toward OXPHOS or alternative nutrient catabolism upon glycolytic blockade. Furthermore, the narrow therapeutic window resulting from shared metabolic dependencies with normal stem cells, coupled with the challenge of penetrating dense tumor stroma or the blood–brain barrier, necessitates more sophisticated pharmacological approaches. Bridging this gap requires transitioning from single-target inhibition to multidimensional synergistic strategies that eliminate metabolic hubs or leverage advanced nanotechnology for spatiotemporally targeted delivery.

The future of CSC eradication lies at the intersection of metabolism and systemic tumor biology. Emerging research must prioritize deconstructing the metabolism-epigenetics crosstalk and the metabolic symbiosis between CSCs and the stromal niche in three-dimensional space. Ultimately, metabolic intervention should be viewed as a catalyst for immunotherapy; by neutralizing the acidic microenvironment through glycolytic inhibition, we can effectively relieve T-cell exhaustion and synergize with immune checkpoint blockade. Such integrative paradigms, focusing on the metabolic resilience of CSCs, will be essential for achieving relapse-free precision oncology and reshaping the future of cancer treatment.

## Figures and Tables

**Table 1:** Association of transcription factors with glycolysis in tumor stem cells.

Transcription Factor	CSC Types	Mechanism	Effect on Glycolysis	Effect on Stemness	Therapeutic Targeting Potential	Reference
HIF-1α	BCSCs, PCSCs, CCSCs	Stabilized by SETD5 under hypoxic conditions; transactivates *CA9*	Upregulation	Enhances self-renewal and chemoresistance	HIF inhibitors (e.g., Flavonoids), CAIX inhibitors	[67,80,81]
c-MYC	GCMSCs, BCSCs, HCSCs, NSCLC	Upregulates *HK2* expression; transcriptionally represses *miR-192-5p*	Promotion	Maintains proliferation and anabolic metabolism	BET inhibitors (e.g., JQ1), MYC-MAX dimerization inhibitors	[82,83]
p53	CSCs, NSCLC	Induces *TIGAR* expression to lower F2,6BP levels	Inhibition	Induces differentiation and apoptosis	MDM2 inhibitors, p53-reactivating small molecules (e.g., APR-246)	[84,85]
NF-κB	CSCs, OCSCs	Mediates inflammatory signaling; enhances glycolytic reprogramming	Promotion	Facilitates EMT and stemness maintenance	IKK inhibitors (e.g., BMS-345541), proteasome inhibitors	[86,87]
ETV4	BCSCs	Promotes expression of glycolytic genes; activates the SHH signaling pathway	Promotion	Maintains stem-like properties	Targeting upstream pathways that activate ETV4	[88]
PGC-1α	CSCs	Activates ERRα and NRF-1/2 to drive OXPHOS; promotes HIF-1α degradation via ROS scavenging	Inhibits (promotes OXPHOS)	Attenuates stemness (promotes differentiation)	PGC-1α modulators	[89,90]
YAP/TAZ	BCSCs, GSCs	Senses ECM mechanical cues; enhances glucose uptake via GLUT1 upregulation	Promotion	Maintains survival in hypoxic environments	YAP inhibitors	[91]

Abbreviations: BCSCs, Breast cancer stem cells; PCSCs, Pancreatic cancer stem cells; CCSCs, Colorectal cancer stem cells; GCMSCs, Gastric cancer mesenchymal stem cells; HCSCs, Hepatocellular carcinoma stem cells; NSCLC, Non-small-cell lung cancer; OCSCs, Ovarian cancer stem cells; GSCs, Glioma stem cells.

**Table 2:** Small-molecule inhibitors and agents targeting key glycolytic enzymes and metabolic pathways in CSCs.

Drug	Structure	Target	Mechanism	Effect	Drug Toxicity	CSCs	Ref.
NHI-1 and NHI-2	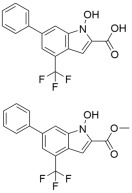	LDH-A	Inhibition of LDH-A, leading to reduced survival rate of GSCs, cell cycle arrest, and apoptosis	Glycolysis was inhibited and the glycolysis rate of GSC was reduced	No specific side effects reported in the study; potential metabolic disturbances due to LDHA inhibition in normal highly glycolytic tissues (e.g., skeletal muscle) are conceivable	GBM cells	[119]
KIS37	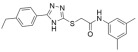	PDK4	Inhibition of PDK4 activity downregulated glycolytic activity and inhibited stem cell markers through the PI3K-Akt-mTOR signaling pathway	Inhibition of pancreatic cancer and colorectal cancer, reduction of stem cell stemness	No specific side effects reported, but potential off-target effects may occur due to pathway inhibition	Pancreatic cancer stem cells, colorectal cancer stem cells	[126]
DCA and SAL	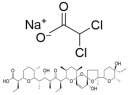	PDK	Not mentioned	Inhibition of PDK activity reduced glycolysis and inhibited lung cancer metastasis and stem cells	No specific side effects reported, but potential metabolic disturbances may arise from PDK inhibition	lung cancer stem cells	[127]
R-2HG	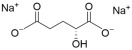	PFKP and FTO	Abrogates FTO/m6A/YTHDF2-mediated post-transcriptional upregulation of PFKP and LDHB, suppressing aerobic glycolysis	Suppresses growth and sensitizes GBM cells to glucose starvation; Inhibits leukemia development	Minimal effect on normal CD34+ hematopoietic stem/progenitor cells (HSPCs), indicating a potential therapeutic window.	GBM cells; leukemia cells	[71]
Citrate	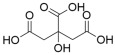	PFK1 and PFK2	Elevated cytosolic citrate inhibits phosphofructokinase activity and glycolysis	Suppress tumor growth, improve drug penetration and boost immunotherapy efficacy.	Low toxicity; potential hypocalcemia and muscle spasms at very high doses (preventable by calcium)	multiple types of tumor cells	[122]
Quercetin	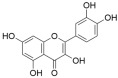	HIF-1a, HK2, GLUT1, LDHA, PFK-1, PDK, PKM2	Inhibits HIF-1α accumulation and synthesis, downregulates major glycolytic enzymes, regulates glycolysis through PI3K/Akt pathway, and triggers ROS generation and ATP depletion.	Suppresses glycolysis; reduces ATP; induces apoptosis; enhances chemo-/radiosensitivity; reverses MDR; inhibits CSC stemness	Low dose: antioxidant; high dose (>50 uM): pro-oxidant, pro-apoptotic	multiple types of tumor cells	[123]
Dodecyl-TPP	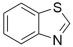	mitochondrial	d-TPP inhibited the growth of CSCs by binding to PDHc and KGDH, blocking TCA cycle, inhibiting OXPHOS and reducing ATP production in CSCs	CSCs were significantly inhibited when combined with glycolysis inhibitors	potential metabolic disturbances from OXPHOS inhibition	breast cancer	[124]

## Data Availability

No new data were created or analyzed in this study.
